# Supplementary material for: Exploring the perceptions of living donation among potential Moroccan donors in the Netherlands
Source: PLoS One. 2025 Aug 1;20(8):e0328212. doi: 10.1371/journal.pone.0328212 (PMC12316208; doi:10.1371/journal.pone.0328212)
Supplement: S1 — (DOCX) [file pone.0328212.s001.docx]

# The online questionnaire.

| Part of the questionnaire | Question | Subject | Outcome |
| --- | --- | --- | --- |
| 1: Demografie  *Demography* | 1 | Hoe oud bent u?  *What is your age?* | Number |
|  | 2 | Wat is uw geslacht?  *What is your sex?* | 1. Man *Man* 2. Vrouw *Woman* 3. Dat zeg ik liever niet *Prefer not to say* |
|  | 3 | Bent u Marokkaans van afkomst?  *Are you of Moroccan descent?* | 1. Ja *Yes* 2. Deels *Partially* 3. Nee *No* |
| 2: Donorstatus  *Donor status* | 1 | Heeft u ooit bloed gedoneerd?  *Have you ever donated blood?* | 1. Ja *Yes* 2. Nee *No* 3. Weet ik niet *I don’t know* |
|  | 2 | Staat u ingeschreven als stamceldonor?  *Are you registered as stem cell donor?* | 1. Ja *Yes* 2. Nee *No* 3. Weet ik niet *I don’t know* |
|  | 3 | Heeft u ooit een orgaan of stuk van uw orgaan gedoneerd?  *Have you ever donated an organ or piece of your organ?* | 1. Ja *Yes* 2. Nee *No* |
| 3: Bereidheid  *Willingness* | 1 | Welke redenen heeft u om wel of niet bloed te doneren?  *What are your reasons to donate blood?* | |
|  | 2 | Welke redenen heeft u om wel of niet stamcellen te doneren?  *What are your reasons to donate stem cells?* | |
|  | 3 | Welke redenen heeft u om wel of niet tijdens uw leven organen zoals een stuk van uw lever of een nier te doneren?  *What are your reasons to donate organs such as a piece of your liver or your kidney during your life?* | |
|  | 4 | Welke informatie heeft u nodig over:  *What information do you need about:*   1. Bloeddonatie *Blood donation* 2. Stamceldonatie *Stem cell donation* 3. Orgaandonatie tijdens het leven *Organ donation during life* | |
|  | 5 | Hoe wilt u informatie over donatie krijgen? Denk hierbij aan informatiebronnen als familie, huisarts, (sociale) media en alles wat verder bij u opkomt.  *How would you like to receive information about donation? Think about information sources such as family, general practitioner, (social) media and everything else that pops your mind.* | |

The original questions in Dutch are reported with the translations to English.
